# Type B Fibers: A Novel Ultrastructural Biomarker for Cognitive Impairment in Neuronal Intranuclear Inclusion Disease

**DOI:** 10.3390/brainsci15101026

**Published:** 2025-09-23

**Authors:** Binbin Zhou, Shaoping Zhong, Yangye Lian, Jingzhen Liang, Luyao Huang, Jing Ding, Xin Wang

**Affiliations:** Department of Neurology, Zhongshan Hospital, Fudan University, 180 Fenglin Road, Shanghai 200032, China; 22111210064@m.fudan.edu.cn (B.Z.); zhong.shaoping@zs-hospital.sh.cn (S.Z.); 22111210062@m.fudan.edu.cn (Y.L.); liang.jingzhen@zs-hospital.sh.cn (J.L.); luyaohuang@fudan.edu.cn (L.H.); ding.jing@zs-hospital.sh.cn (J.D.)

**Keywords:** neuronal intranuclear inclusion disease, GGC repeat expansion, cognitive impairment, ultrastructural pathology, MMSE score

## Abstract

Background/Objective: Neuronal intranuclear inclusion disease (NIID) is characterized by widespread deposition of eosinophilic intranuclear inclusions in multiple systems throughout the body. The aim of this study was to investigate the clinical and phenotypic features of NIID, with a focus on the potential association between the morphological features of fibrils formed by polyG (polyglycine) proteins and cognitive dysfunction in patients with NIID. Methods: This study involved a retrospective collection of clinical data from 15 patients with NIID harboring GGC repeat expansions in the *NOTCH2NLC* (Notch 2 N-Terminal Like C) gene (including symptoms, signs, biochemical markers, cranial MRI, MMSE, and MoCA cognitive scores). All patients underwent skin biopsy, with one additional autopsy of brain tissue. Some skin samples were stained with hematoxylin and eosin (H&E) and immunohistochemistry (IHC) staining with anti-p62 antibody. The remaining skin samples and brain tissue samples obtained from autopsies were analyzed using anti-p62 antibody immunofluorescence (IF) staining and transmission electron microscopy (TEM). The number of GGC repeats was quantified using repeat primer PCR (RP-PCR). Based on ultrastructural characteristics (morphology and diameter), inclusion fibers were classified into two subtypes, and differences in the severity of cognitive impairment between subtypes were compared. Results: The majority of patients in this cohort with NIID were female (73.3%), with an average age of onset of 61.06 ± 7.67 years. The core clinical manifestations were cognitive decline (93.3%) and autonomic dysfunction (93.3%). Cranial MRI revealed characteristic DWI “ribbon sign” in 93.3% of patients, accompanied by lateral ventricle enlargement (93.3%), cerebellar atrophy (86.6%), and high T2-FLAIR signal in the corpus callosum (93.3%). All patients were found to have pathogenic GGC amplification in the *NOTCH2NLC* gene (median 115, range 88–210). Skin/brain tissue pathology confirmed p62-positive nuclear inclusions, and transmission electron microscopy revealed two fiber subtypes for the first time: type A (Long, thin filamentous, 202.38 ± 42.35 nm) and type B (short rod-shaped, 73.08 ± 11.56 nm). Group analysis indicated that the diameter of fibers was significantly larger in the cognitive impairment group (*p* < 0.05), and the type B fiber group had lower cognitive levels (*p* < 0.05) and larger diameters (*p* < 0.05), suggesting a strong association between type B fibers and severe cognitive impairment and poor prognosis. Conclusions: The presence of two different forms of fibrils, type A and type B, in the inclusion bodies of NIID patients, and the poorer cognitive level of NIID patients in the type B group than that of type A suggest that type B fibrils can be used as a novel pathological marker of severe cognitive impairment and poor prognosis in NIID.

## 1. Introduction

Neuronal Intranuclear Inclusion Disease (NIID) is a neurodegenerative disease caused by an aberrant amplification of the GGC repeat sequence in the 5′untranslated region (5′UTR) of the *NOTCH2NLC* (Notch 2 N-Terminal Like C) gene [1,2,3,4]. The core pathological hallmark is the extensive deposition of eosinophilic intranuclear inclusion bodies in neurons, glial cells, and peripheral tissues such as skin [4,5,6]. These inclusion bodies are formed by aggregates of polyglycine (polyG) proteins produced by aberrant translation, which ultimately lead to neuronal dysfunction by disrupting nuclear membrane integrity and interfering with nucleoplasmic transport [7,8,9]. Clinical manifestations of NIID are highly heterogeneous and can involve the central, peripheral, and autonomic nervous systems. The most common manifestations are cognitive impairment and autonomic dysfunction, followed by episodic symptoms, movement disorder, and muscle weakness [10,11,12]. Animal model studies have shown that aberrant aggregation of polyG proteins induces microglia-mediated neuroinflammation, stress granule formation, and whole-cell translational inhibition [13,14,15], which may underlie the molecular basis of cognitive impairment. Notably, transmission electron microscopy revealed the existence of different morphologies of fibrillar structures in polyG inclusion bodies [16], but whether the morphological differences of fibrils in these ultrastructures are directly correlated with the severity of cognitive impairment still lacks systematic studies.

Available evidence suggests that the physical properties of polyG protein aggregates in NIID may influence their pathogenicity. For example, polyG proteins resulting from GGC repeat amplification are prone to phase separation and form inclusion bodies with different aggregation states [9]. Pathological processes, such as kernel stress and impaired ribosome biosynthesis, are closely associated with conformational changes of polyG aggregates [13]. These findings suggest that morphological features of inclusion body fibrils may serve as biomarkers reflecting disease progression. However, no study to date has quantitatively typed the morphology of fibrils and clarified its association with the cognitive prognosis of patients. In this study, we used transmission electron microscopy to classify inclusion body fibrils into two subtypes, type A (long thin filaments) and type B (short rods), based on their ultrastructural features, and investigated the potential association between different morphologies and the severity of cognitive dysfunction, with the aim of providing new insights into disease mechanisms and clinical interventions.

## 2. Methods

### 2.1. Study Participants

This study retrospectively analyzed patients with adult-onset neuronal intranuclear inclusion body disease (NIID) who were seen between January 2022 and June 2024 at Zhongshan Hospital, Fudan University. Patients were examined by at least 2 experienced neurologists and were included in the study only if the following inclusion criteria were met: (1) patients exhibited clinical symptoms of NIID as previously described in the literature [1,17]; (2) cranial MRI was required to show that patients had extensive and symmetrical cerebral white matter damage; (3) genetic testing was required to confirm that the GGC trinucleotide repeat of the *NOTCH2NLC* gene was GGC trinucleotide repeats of the *NOTCH2NLC* gene were amplified more than 66 times; (4) Skin pathology examination needed to find p62-positive intranuclear inclusion bodies, and the intranuclear inclusion bodies were observed under the skin electron microscope. Only those who fulfilled the above four conditions could be included in the study. The exclusion criteria included: first, genetic testing revealed that the CGG trinucleotide repeat amplification of the *FMR1* (Fragile X Messenger Ribonucleoprotein 1) gene was more than 55 times; and second, suffering from acquired or inherited metabolic cerebral leukoencephalopathy caused by other clear etiologies.

All tissue samples were obtained after the patients signed a written consent form in accordance with the Chinese bioethics law and the Declaration of Helsinki. The study was approved by the Ethics Committee of Zhongshan Hospital, Fudan University.

### 2.2. Clinical Assessment

Retrospective data collection was performed, including age, gender, number of GGC repeats, first symptom, duration of disease, clinical symptoms, blood pressure, glomerular filtration rate, deep white matter Fazekas score, total cholesterol, triglycerides, low-density lipoproteins, high-density lipoproteins, lactic acid, C-reactive protein, leukocyte counts, the Brief Mental State Examination (MMSE), Montreal Cognitive Assessment Scale (MoCA), fiber length and diameter, and other detailed clinical information. All measurements were performed by trained laboratory technicians and examiners.

### 2.3. Neuroimaging

All participants underwent routine brain MRI using a 3.0 T or 1.5 T MR scanner, and images were individually evaluated by at least 2 neuroimaging specialists who were unaware of the clinical information. The extent of white matter and brain atrophy on T2WI sequences was graded according to previous studies, and white matter lesion volume measurements were performed manually using ITK-SNAP software (version 4.4.0) [18].

### 2.4. Genetic Testing

The venous blood of all subjects was centrifuged to extract the genomic DNA of the subjects using the Tengen Gene Extraction Kit (TianGen Biotech Co., Ltd. Beijing, China), and the GGC repeat amplified fragments of the *NOTCH2NLC* gene were detected using the repeat-primed PCR (RP-PCR) method according to the methods previously reported in the literature [3,19]. The three primers were as follows: NOTCH2NLC-F: 5′-FAM-GTGCTTCGGACCGTAGCGCCAGG-3′; NOTCH2NLC-R: 5′-TGCTGTGCACGCTGTAGTGAGCCGATGATA-3′; and NOTCH2NLC-linker-R: 5′-ACAGCGCCCCTCAGCCCCATACT-3′. The following thermal conditions were applied: initial denaturation at 94 °C for 3 min, followed by 35 cycles of 94 °C for 30 s, 55 °C for 30 s, and 73 °C for 20 s, concluding with an extension at 72 °C for 10 min. The GGC repeat amplified fragments of the *NOTCH2NLC* gene were identified by the 3700xl Genetic Analyzer (Thermo Fisher Scientific, Waltham, MA, USA). The products of RP-PCR were identified by capillary electrophoresis, and the data were processed with GeneMapper software (version 2.6.3) to analyze the fragment size and number of repeats. The presence of a sawtooth tail pattern in the electropherogram was regarded as evidence of a disease-associated repeat expansion.

*FMR1* gene GGC repeat expansion detection was performed by the RP-PCR method with reference to the previous literature [20].

### 2.5. Pathological Examination of Brain Autopsy and Skin Biopsy

Skin biopsy was performed after informed consent. After local anesthesia with lidocaine, skin biopsy specimens were obtained from the distal calf (10 cm above the external ankle) of 15 patients. One portion of the skin tissue was fixed with 2% glutaraldehyde at 4 °C overnight for subsequent transmission electron microscopy analysis. The remaining portion of skin tissue was fixed with 10% formalin and then embedded in paraffin, cut into 6 μm thick, and the paraffin sections were stained with hematoxylin and eosin (H&E) and immunohistochemically stained with anti-p62 (1:200; 18420-1-AP, Proteintech) antibody [9,21].

Following the death of one patient, brain removal was performed with the consent of the next of kin, in accordance with the local research review committee (Human Research Committee of Zhongshan Hospital, Fudan University) and the 1995 Declaration of Helsinki (as amended in Edinburgh, 2000). Rapid localization of the parietal lobe cortex and post-central gyrus was achieved by identifying the central sulcus, parieto-occipital sulcus, and postcentral sulcus (the apex of the post-central gyrus is typically located approximately 1.0 cm posterior to the intersection of the eyebrow-occipital line and the anterior hairline). Following brain tissue resection, one portion was fixed in 10% formalin, embedded in paraffin, sectioned at 6 μm thickness, and subjected to immunofluorescence staining with the p62 antibody (1:500; 18420-1-AP, Proteintech) [9,21]. The remaining portion was fixed in 2.5% glutaraldehyde for subsequent transmission electron microscopy analysis.

### 2.6. H&E Staining

Take out the skin paraffin sections and heat at 60 °C for 2 h. Dewax with xylene for 5 min, hydrate with ethanol in a gradient (2 min each), and rinse with tap water for 2 min. Hematoxylin stain for 2 min, then rinse with tap water until no dye is eluted. Remove excess dye by immersion in hydrochloric acid ethanol. Rinse with tap water for 1 min, counterstain with saturated lithium carbonate solution for 4 times, rinse with tap water for 2 min. Eosin stain for 30 s, rinse with tap water for 30 s. Dehydrate with graded ethanol solutions for 2 min each, followed by 3 times with absolute ethanol for 2 min each. Then treat with xylene for 5 min to achieve transparency. Finally, mount with neutral resin, observe, and photograph.

### 2.7. Immunohistochemistry and Immunofluorescence Staining

Immunohistochemistry and immunofluorescence staining maintain consistency in the sample pretreatment steps. Skin paraffin sections must undergo baking, dewaxing, and hydration, and the operational procedure is the same as that of conventional HE staining. In immunohistochemical staining, the sections were washed with PBS three times for 2 min each, then incubated with 3% hydrogen peroxide solution at room temperature for 5 to 10 min to eliminate endogenous peroxidase activity. The sections were placed in citrate buffer (pH 6.0) and subjected to heat retrieval at 95 °C in a water bath for 10 min, followed by natural cooling to room temperature. Then, they were incubated with PBS containing 5% donkey serum at room temperature for 2 h. After that, 1:200 diluted rabbit anti-p62 antibody (18420-1-AP, Proteintech) was added and incubated overnight at 4 °C. After washing with PBS three times for 10 min each, 1:500 diluted horseradish peroxidase-conjugated donkey anti-rabbit antibody (711035152, Jackson ImmunoResearch, Lancaster, PA, USA) was added and incubated at room temperature for 1 h. After washing with PBS three times for 10 min each again, the DAB immunohistochemical coloring reagent kit (E670033, Shengong Biotechnology Co., Ltd., Shanghai, China) was used for the color development reaction under dark conditions at room temperature. Specific operations were referred to in the kit instructions. After thorough washing with distilled water, the sections were stained with hematoxylin following steps similar to HE staining, and finally mounted with neutral gum for observation and photography under a microscope.

In immunofluorescence staining, the sections were permeabilized with 0.5% SDS-TBS solution for 10 min after antigen retrieval. A TBS blocking solution containing 5% donkey serum was used to incubate at room temperature for 2 h. Rabbit anti-p62 polyclonal antibody (18420-1-AP, Proteintech) was diluted in blocking solution at a ratio of 1:500 and incubated overnight at 4 °C. After washing with TBS 3 times for 10 min each time, a 1:500 diluted fluorescently labeled donkey anti-rabbit secondary antibody (Jackson ImmunoResearch, Lancaster, PA, USA) was added and incubated in the dark at room temperature for 1 h. After one TBS wash, the cell nuclei were counterstained with DAPI for 5 min, followed by 3 additional TBS washes of 10 min each. Finally, the sections were mounted and observed under a fluorescence microscope to collect images.

### 2.8. Transmission Electron Microscope

The preparation and observation process of electron microscopy specimens primarily involves specimen preparation, ultrathin sectioning, staining, and electron microscopy observation. First, skin and brain autopsy (parietal lobe cortex and post-central gyrus) tissues were cut into small blocks measuring approximately 1 mm × 1 mm × 3 mm. These were pre-fixed in 2.5% glutaraldehyde solution, rinsed in phosphate-buffered saline (pH 7.3), and then post-fixed in 1% osmium tetroxide. Subsequently, samples underwent dehydration with graded concentrations of acetone, resin permeation, and embedding. Ultra-thin sections (50–100 nm) were prepared using a Leica microtome and diamond blades. Sections underwent double electron staining with 3% uranyl acetate and lead nitrate. Submicroscopic structures, including nuclear inclusions, were observed using a Hitachi HT-7700 transmission electron microscope (Hitachi High-Technologies Corporation, Tokyo, Japan), with images captured and recorded [22,23]. Given the importance of detecting nuclear inclusions, all samples underwent two independent ultrastructural examinations to enhance the positive detection rate.

### 2.9. Statistical Analysis

SPSS 26.0 software was used for statistical analysis. Measurements that conformed to normal distribution were expressed as mean ± standard deviation, and comparisons between groups were made using the independent samples *t* test; non-normally distributed measurements were expressed as median (interquartile spacing), and comparisons between groups were made using the Mann–Whitney U test. Categorical variables were described as frequencies (percentages), and comparisons of differences between groups were made using the χ^2^ test or Fisher’s exact test (when theoretical frequencies were <5). Between-group correlations for variables related to cognitive dysfunction were analyzed by Spearman’s rank correlation. All statistical tests were two-sided, and *p* < 0.05 was considered a statistically significant difference.

## 3. Results

### 3.1. Demographics and Clinical Features

**Figure 1:**
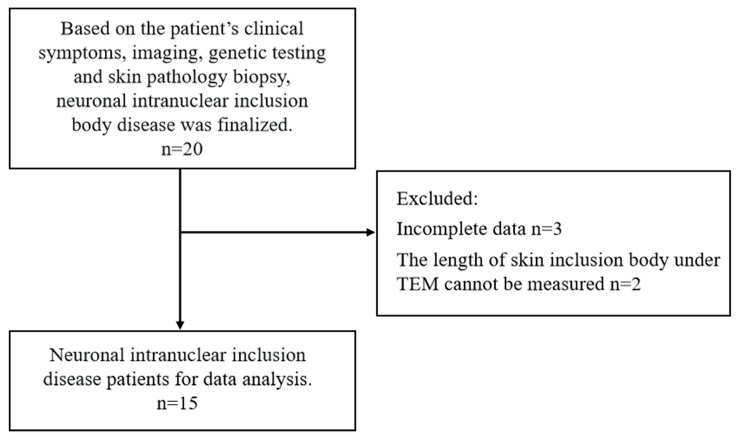
Flow chart for inclusion and exclusion of study participants. TEM, Transmission electron microscope.

According to the screening criteria for adult-type NIID enrollment, a total of 20 patients were included in this study; of these, 2 were lost to follow-up, 3 were excluded due to incomplete data, and 15 were finally included for the analysis (Figure 1). Of the 15 patients, 4 (26.6%) were males and 11 (73.3%) were females; the mean age of onset was (61.06 ± 7.67) years; the mean time from onset to diagnosis ranged from 3 to 9 years; and 7 cases (46.6%) had a family history and 8 (53.3%) were disseminated cases. The clinical manifestations of the patients were highly heterogeneous, with damage to the central nervous system (CNS) and other systems predominating. The central nervous system damage mainly included cognitive impairment (93.3%), autonomic dysfunction (93.3%), tremor (53.3%), unsteady walking (53.3%), limb weakness (20.0%), and sensory deficits (20.0%). Some patients had seizure symptoms, including episodic headache (53.3%), encephalitis-like seizure (33.3%), stroke-like seizure (6.6%), epileptic seizure (26.6%), and seizure mental abnormality (20.0%). In addition, 20.0% of the patients had a history of diabetes mellitus (Table 1).

### 3.2. Neuroradiologic Findings

**Figure 2:**
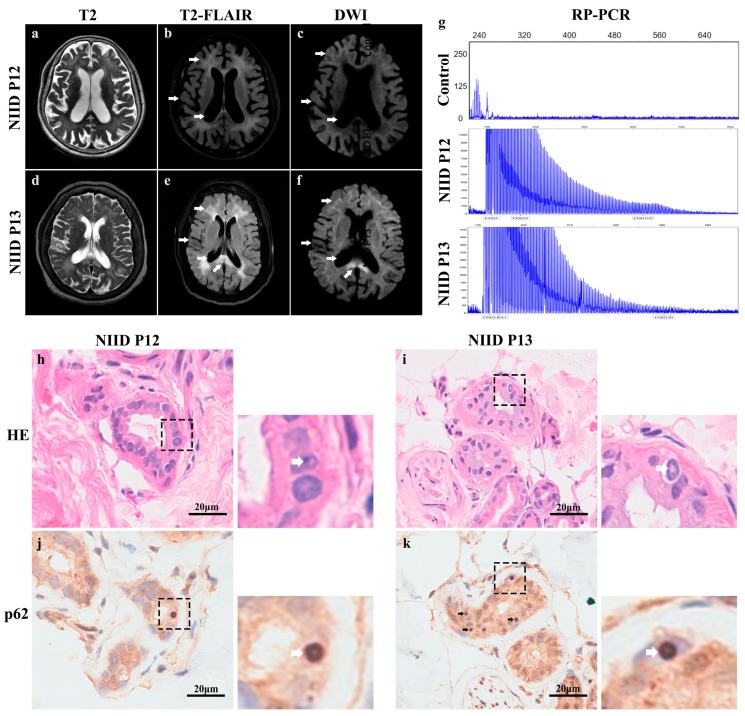
Neuroimaging, genetic and histopathologic features of patients with NIID. (**a**–**c**) Cranial MRI (Magnetic Resonance Imaging) of patient 12 shows extensive white matter lesions at the corticomedullary junction, with the corresponding areas showing high signal on DWI sequences and concomitant brain atrophy. (**d**–**f**) Cranial MRI of the 13th patient also showed extensive white matter lesions at the corticomedullary junction, and in addition, the corpus callosum compression area showed high signal on DWI sequences with concomitant brain atrophy and ventricular dilatation. (**a**–**f**) White arrows indicate: upper arrow—subcortical white matter lesion; middle arrow—brain atrophy feature; lower arrow—lateral ventricular enlargement (**b**,**c**,**e**,**f**) and DWI high signal in the corpus callosum pressure area (**f**). (**g**) Repeat Primer Polymerase Chain Reaction (RP-PCR) electrophoretic peaks: control samples show a single peak; two samples from patients with NIID (patients 12 and 13) show a characteristic stepwise decreasing peak pattern suggesting repeated amplification of the GGC (repetitions of 110 and 120, respectively). (**h**,**i**) Hematoxylin-eosin (H&E) staining of skin tissues from patient 12 (**h**) and patient 13 (**i**): eosinophilic inclusion bodies are seen in the nuclei of the epithelial cells of the sweat ducts (dashed area; magnification of the corresponding area is shown on the right, The white arrow indicates the inclusion body). (**j**,**k**) Immunohistochemical staining for p62 in skin tissues of patient 12 (**j**) and patient 13 (**k**): p62-positive inclusion bodies are seen in the nuclei of sweat gland duct epithelial cells (black dashed area; enlargement of the corresponding area is shown on the right, The white arrow indicates the inclusion body). Scale bar: 20 μm (**h**–**k**); original magnification: ×40 (**h**–**k**). NIID = neuronal intranuclear inclusion disease; H&E = hematoxylin and eosin; DWI = diffusion-weighted imaging; T2 = T2-weighted imaging; T2 FLAIR = T2 fluid-attenuated inversion recovery; MRI = magnetic resonance imaging; P12 = Patient 12; P13 = Patient 13; p62 = Sequestosome 1.

The cranial MRI results of 15 patients with NIID showed that 14 cases (93.3%) had characteristic high signal changes at the ependymal junction on DWI (commonly known as the “ribbon sign” or “diaper sign”); 14 cases (93.3%) had enlarged lateral ventricles; cerebellar atrophy was present in 13 cases (86.6%); T2 FLAIR high signal in the centrum semiovale was present in 14 cases (93.3%); T2 FLAIR high signal in the brainstem was present in 9 cases (60.0%); T2 FLAIR high signal in the corpus callosum was present in 14 cases (93.3%); T2 FLAIR high signal in the exocapsular capsule was present in 11 cases (73.3%); and T2 FLAIR high signal in the internal capsule was present in 2 cases (13.3%). Moreover, 1 case (6.6%) had striatum T2 FLAIR high signal; 4 cases (26.6%) had middle cerebellar peduncle (MCP) T2 FLAIR high signal; and 7 cases (46.6%) had cerebellar vermis T2 FLAIR high signal. Here we show typical images of 2 of these patients (NIID P12, P13) (Figure 2a–f). Taken together, the above imaging characterization showed that white matter lesions in the centrum semiovale, corpus callosum, and brainstem with cerebellar atrophy and enlarged ventricles were prevalent in our patients, suggesting that these may be common manifestations of cerebral white matter lesions in patients with NIID.

### 3.3. Genetic Analysis

All subjects were tested for CGG repeat amplification in the *FMR1* and *NOTCH2NLC* genes by Repeat Primer PCR (RP-PCR). The results showed that all subjects were negative for amplification of CGG repeats in the FMR1 gene. significant amplification of GGC repeats in the *NOTCH2NLC* gene was detected in all 15 NIID patients (≥60 repeats), and the number of GGC repeats in all NIID patients ranged from 88 to 210, with a median of 115 repeats (range: 100–148) (Figure 2g).

### 3.4. Dermatopathological Staining and Electron Microscopy

**Figure 3:**
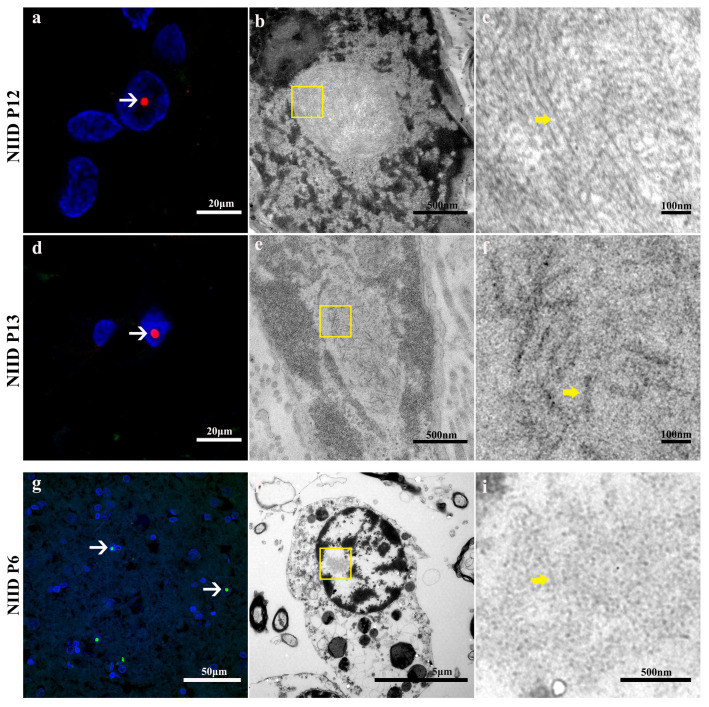
Two morphologically distinct fiber structures are present in the skin and autopsy brain tissue of NIID patients. (**a**) Immunofluorescence staining of skin tissue from patient 12 reveals p62-positive intranuclear inclusion bodies deposits (indicated by white arrows). scale bars: 20 μm. (**b**,**c**) Transmission electron microscopy of patient 12 skin tissue: (**b**) Inclusion body deposition in the nucleus of fibroblasts (the area highlighted in the yellow box); (**c**) High magnification of inclusion bodies showing that they are composed of elongated fibrous filaments (type A, indicated by yellow arrows). Scale bar: 500 nm (**b**); 100 nm (**c**). (**d**) Immunofluorescence staining of skin tissue from patient 13 reveals p62-positive intranuclear inclusion bodies deposits (indicated by white arrows). Scale bar: 20 μm. (**e**,**f**) Transmission electron microscopy of patient 13 skin tissue: (**e**) eosinophilic intranuclear inclusion body deposits in fibroblasts (the area highlighted in the yellow box); (**f**) high magnification of inclusion bodies showing that they are composed of short, rod-shaped fibrils (type B, indicated by yellow arrows). Scale bar: 500 nm (**e**); 100 nm (**f**). (**g**) Immunofluorescence staining of brain tissue (post-central gyrus) from the autopsy of patient 6 reveals p62-positive intranuclear inclusion deposits (indicated by white arrows). Scale bar: 50 μm. (**h**,**i**) Transmission electron microscopy of autopsy brain tissue from patient 6: (**h**) eosinophilic inclusion bodies in the nucleus of astrocytes (the area highlighted in the yellow box); (**i**) High magnification of inclusion bodies showing that they are composed of short rod-shaped fibrils (type B, indicated by yellow arrows). Scale bar: 5 μm (**h**); 500 nm (**i**).

We performed skin biopsies on all 15 patients and obtained autopsy brain tissue specimens from one of them. Hematoxylin-eosin (HE) staining of the skin tissues of all patients showed the presence of rounded eosinophilic intranuclear inclusions in the nuclei of sweat gland duct epithelial cells and fibroblasts (Figure 2h,i); immunohistochemistry and immunofluorescence staining further confirmed the presence of p62-positive intranuclear inclusions in the duct epithelial cells of the lesser sweat glands and fibroblasts (Figure 2j,k and Figure 3a,d). The patient’s skin was basically structurally normal, with no subcutaneous fat atrophy, inflammatory cell/lymphocyte infiltration, or significant dermal edema. Transmission electron microscopy (TEM) examination of skin tissue revealed two different morphologies of eosinophilic inclusions in the nuclei of fibroblasts (Figure 3b,c,e,f). In the autopsy pathology of the brain tissue of the 6th patient with NIID, immunofluorescence staining revealed the presence of p62-positive intranuclear inclusion bodies in the post-central gyrus (Figure 3g), while TEM observation revealed the presence of eosinophilic intranuclear inclusion bodies in astrocytes (Figure 3h,i), and the morphological characteristics of the brain tissue inclusions in patient 6 are similar to those of the skin tissue inclusions in patient 13. In addition, analysis of the inclusion bodies in all patients revealed the presence of two morphological types with differences in the diameter of the inclusion body filaments; measurements of the inclusion body filaments showed an average length of (167.91 ± 69.37) nm and an average diameter of (12.05 ± 5.47) nm, with long, thin, filamentous, convoluted shapes defined as type A (Figure 3c), and relatively thick, short, rod-like shapes as type B (Figure 3f,i).

### 3.5. Comparison of Clinical Features Between NIID Patients with and Without Cognitive Impairment

The overall cognitive level of the patients was assessed using the Brief Mental State Examination Scale (MMSE Scale). All patients had a middle school level of education. Subjects were categorized into two groups based on MMSE scores: scores ≥ 24 were categorized as the no cognitive dysfunction group, and scores < 24 were categorized as the cognitive dysfunction group. Statistical analysis showed that in terms of clinical symptoms, no significant differences were observed between the two groups in terms of the incidence of unsteady walking and autonomic dysfunction. Encephalitis-like episodes were seen only in the cognitive dysfunction group (5 cases, 55.5%), while they did not occur in the group without cognitive dysfunction, and the difference between the groups approached the level of significance (*p* = 0.093). In addition, the prevalence of cognitive dysfunction was higher in patients with comorbid diabetes (33.3%) (Table 2).

Comparison of cranial MRI performance showed no significant difference between the two groups in terms of the incidence of DWI high signal at the dermatomedullary junction, ventricular enlargement, and cerebellar atrophy. The incidence of T2 FLAIR high signal in the centrum semiovale was 100% in the cognitive dysfunction group, which was significantly higher than that in the group without cognitive dysfunction (83.3%). However, the incidence of internal capsule and striatum T2 FLAIR high signal was higher in the group without cognitive dysfunction than in the group with cognitive dysfunction. There was no significant difference in white matter lesion volume between the two groups (Table 2). Comparison of biochemical parameters showed no significant differences in total cholesterol, triglycerides, low-density lipoprotein, high-density lipoprotein, lactate, and white blood cell count between the two groups. The mean serum C-reactive protein level was higher in the cognitive dysfunction group (8.73 ± 11.9 mg/L) than in the group without cognitive dysfunction, and the difference approached the level of significance (*p* = 0.089) (Table 2). Comparison of fiber filament morphology revealed that the mean length of fiber filaments in the cognitive dysfunction group was lower than that of the group without cognitive dysfunction, with the difference approaching the level of significance (*p* = 0.081). The mean diameter of fibrils in the cognitive dysfunction group was significantly greater than that in the group without cognitive dysfunction (*p* < 0.05) (Table 2). In conclusion, elevated serum C-reactive protein levels, shortened fibrillar length, and thickened fibrillar diameter may be associated with cognitive dysfunction.

### 3.6. The Clinical Prognosis of NIID Patients in the Type B Fiber Group Was Worse than That of the Type A Group

By measuring and morphologically classifying the inclusion body fibrils observed by skin tissue electron microscopy, they were divided into two types: type A (long, thin filaments) and type B (short rods). Statistical analysis showed that no significant differences were observed between patients with type A and type B in terms of disease duration, number of GGC repeats, and white matter lesion volume. However, in terms of cognitive function assessment (MMSE scores, MoCA scores), patients in the type B group had significantly lower scores than those in the type A group (the difference was statistically significant). Fibrillar morphology measurements showed that the mean length of fibrils in patients in the type B group was significantly shorter than that in the type A group, whereas the mean diameter of fibrils was significantly larger than that in the type A group (the difference was statistically significant) (Table 3). In summary, compared with the cognitive scores of the normal population, both groups of patients included in this study had lower cognitive scores, with a more pronounced decline in the type B group. These findings suggest that two different morphologies of inclusion body fibrils, especially type B, may be associated with cognitive dysfunction and that type B may portend a poor prognosis for clinical patients.

## 4. Discussion

Neuronal Intranuclear Inclusion Disease (NIID) is a neurodegenerative disease characterized by abnormal protein aggregation in the nucleus of neurons, and its typical clinical manifestations include progressive cognitive deficits, motor impairments, and autonomic dysfunction [2,17,24]. The disease is highly clinically heterogeneous, and current diagnosis relies on the detection of p62-positive intranuclear inclusion bodies in skin biopsies [21,25], but lacks effective prognostic indicators. Existing studies have focused on the association between the length of GGC repeat amplification in the 5′UTR region of the *NOTCH2NLC* gene and clinical phenotypes [26,27,28], while the pathological significance of the ultrastructural features of inclusion bodies is poorly understood, which greatly limits the development of disease typing and precise intervention strategies. In this study, two ultrastructural subtypes of inclusion body fibrils, type A (long thin filaments) and type B (short rods), were systematically revealed in NIID patients by integrating transmission electron microscopy (TEM) ultrastructural analyses, genetic testing, and neuropsychological assessment, and type B fibrils were significantly associated with more severe cognitive dysfunction. This finding suggests that the morphological characteristics of the fibrils may influence the disease progression trajectory and provides a new pathomechanistic framework for resolving the clinical heterogeneity of NIID.

In this study, we retrospectively analyzed the clinical characteristics of 15 patients with adult-type neuronal intranuclear inclusion disease (NIID). The results showed that the mean age of onset of the patients was (61.06 ± 7.67) years, and the median duration of the disease was 4 years (3–9 years), a result that is generally consistent with previous reports in the literature [3,4,29]. The spectrum of neurological symptoms of NIID is known to include cognitive impairment, limb weakness, seizure encephalopathy, tremor, stroke-like episodes, ataxia, myopathy, peripheral neuropathy, and autonomic dysfunction [1,3,5,19]. It is worth noting that in addition to the central nervous system, NIID can involve multiple systems such as respiratory, urinary, motor and digestive systems, presenting a highly heterogeneous clinical picture. In this cohort, the neurological symptoms were mainly cognitive impairment (93.3%), which is in line with the results previously reported in the literature [5]; the secondary manifestations were autonomic dysfunction (e.g., urinary retention, vomiting, etc.), a phenomenon that has been reported in both domestic and international studies [4,11,30,31], which further corroborates the multifaceted nature of the clinical manifestations of NIID.

In this study, cranial MRI results showed the presence of characteristic high signal in the corticomedullary junction area of DWI sequences in 14 patients (93.3%), a finding consistent with previous reports [1]. The incidence of T2-FLAIR high signal in the centrum semiovale and corpus callosum was similarly high at 93.3%, a feature that has been reported in both the national and international literature [32,33,34,35]. It is worth noting that several retrospective studies have confirmed that abnormal signals in the corticomedullary junction and corpus callosum pressure can be used as early imaging markers of cognitive dysfunction [6,33,36,37], suggesting that clinical attention should be focused on these regions to improve the early diagnosis of NIID. In addition, some patients presented cerebellar-related abnormalities: cerebellar atrophy (86.6%, 13/15), cerebellar vermis high signal (46.6%, 7/15), and middle cerebellar peduncle high signal (26.6%, 4/15), a distribution pattern consistent with that reported by Sugiyama et al. [38]. The presence of enlarged lateral ventricles in all cases, some of which were accompanied by signs of hydrocephalus, suggests that hydrocephalus should be included in the NIID clinical screening and evaluation system to improve the diagnostic rate.

Previous studies have shown that elevated neutrophil/lymphocyte ratio (NLR) is significantly associated with neuronal intranuclear inclusion disease (NIID) [39]; cerebrospinal fluid p-Tau181 protein is also significantly elevated in NIID patients [40]. In addition, plasma neurofilament light chain (NfL) levels were significantly elevated in patients with NIID compared to healthy controls, suggesting that NfL levels may serve as a potential biomarker for NIID [41]. In this study, it was further found that there were no statistical differences between the cognitive dysfunction group and the group without cognitive impairment in total cholesterol, triglycerides, LDL, HDL, lactate, and leukocyte counts; however, serum C-reactive protein (CRP) levels in the cognitive dysfunction group were significantly higher than those in the control group (*p* = 0.089), suggesting that CRP may serve as a NIID-related cognitive impairment early biomarkers, a result that suggests that inflammatory responses may be involved in the pathogenesis of NIID.

Currently, the central pathological basis for skin biopsy to confirm the diagnosis of adult-onset neuronal intranuclear inclusion disease (NIID) is the presence of characteristic intranuclear inclusion bodies [21]. Dermatopathological examination of all 15 patients in this study showed the presence of typical p62-positive intranuclear inclusion bodies in sweat gland duct epithelial cells and fibroblasts, with a detection rate of 100%, which is consistent with previous reports [25]. Electron microscopic observation further confirmed that these inclusion bodies consisted of filamentous material without membrane structure, consistent with the ultrastructural features described in the literature [23,42]. Notably, the present study identified two morphologically distinct subtypes of inclusion body fibrils (type A and type B) in patients with NIID, the formation mechanism of which may be related to the amyloid self-assembly process [43,44,45,46]. By intergroup comparison of the severity of cognitive impairment, patients carrying short rod-like structures (type B) fibrils showed more significant cognitive dysfunction. This finding suggests that different morphological types of fibrils may mediate neurotoxicity and participate in the pathogenic process of NIID by causing conformational changes through dynamic assembly.

The cross-sectional design of this study also has some limitations. First, the rare disease characteristics of NIID and the invasive nature of skin biopsy resulted in the inclusion of a small sample size, which may affect statistical validity. Furthermore, the high proportion of female participants in this study may introduce gender selection bias, which limits the generalizability of the findings to some extent. Subsequent research urgently requires expanding sample sizes and consciously balancing gender ratios to validate these findings and further explore the specific mechanisms by which gender influences NIID. Additionally, due to the limited sample size of the type B fibrillar group, there may be an increased risk of Type I errors to some extent. Future studies should expand the sample size, particularly for the type B fibrillar patient cohort, to validate the preliminary findings of this research and further evaluate its potential as a biomarker. Second, the cross-sectional design limited our interpretation of the dynamic evolution of fibrillar morphology and its association with longitudinal decline in cognitive function, and long-term follow-up data need to be included in the future to verify whether type B fibrillar morphology indeed predicts faster disease progression. Finally, despite the ultrastructural differences clarified by electron microscopy, mechanistic studies such as proteomics or cellular models are lacking to elucidate the molecular basis of the formation of fibrils with different morphologies and their specific effects on neuronal function.

## 5. Conclusions

In summary, the present study found a significant association between the morphology of inclusion body fiber filaments (type A/B) and the degree of cognitive impairment in patients with NOTCH2NLC-associated NIID, which provides a new pathological basis for the stratification of disease subtypes. This finding suggests that fiber filament ultrastructural features may reflect different neurodegenerative mechanisms, and future studies should expand the samples and integrate multi-omics analyses to elucidate the molecular basis of the morphological differences and their clinical translational value, ultimately laying the foundation for precise interventions.

## Figures and Tables

**Table 1:** Demographic and Clinical features of Patients with NOTCH2NLC-Related Neuronal Intranuclear Inclusion Disease (NIID).

Characteristic	Total
Number	15
Female, *n* (%)	11 (73.4%)
Age at onset (y), mean ± SD	61.06 ± 7.67
Disease duration (y), mean (range)	4 (3–9)
Positive family history, *n* (%)	7/15 (46.7%)
**Clinical manifestation**	
Cognitive impairment, *n* (%)	14/15 (93.3%)
Tremor, *n* (%)	8/15 (53.3%)
Gait disturbance, *n* (%)	8/15 (53.3%)
Abnormal behavior, *n* (%)	3/15 (20%)
Encephalitis-like episode, *n* (%)	5/15 (33.3%)
Epileptic episode, *n* (%)	4/15 (26.6%)
MELAS-like episode, *n* (%)	1/15 (6.6%)
Autonomic dysfunction, *n* (%)	14/15 (93.3%)
Limb weakness, *n* (%)	3/15 (20%)
Sensory disturbance, *n* (%)	3/15 (20%)
Headache, *n* (%)	8/15 (53.3%)
Diabetes, *n* (%)	3/15 (20%)
**Imaging manifestations**	
DWI high signal at dermatomedullary junction, *n* (%)	14/15 (93.3%)
T2 high signal in corpus callosum, *n* (%)	14/15 (93.3%)
T2 high signal of the external capsule, *n* (%)	11/15 (73.3%)
Inner capsule T2 high signal, *n* (%)	2/15 (13.3%)
Striatum T2 high signal, *n* (%)	1/15 (6.6%)
T2 high signal in thalamus, *n* (%)	5/15 (33.3%)
T2 high signal in brainstem, *n* (%)	9/15 (60%)
T2 high signal in the middle cerebellar peduncle, *n* (%)	4/15 (26.6%)
T2 high signal in the cerebellum, *n* (%)	7/15 (46.6%)
T2 high signal in the centrum semiovale, *n* (%)	9/15 (60%)
Lateral ventricle enlargement, *n* (%)	14/15 (93.3%)
Cerebellar atrophy, *n* (%)	13/15 (86.6%)
White matter lesion volume (cm^3^), mean ± SD	90.07 ± 33.71
**Blood biochemical examination**	
Glomerular filtration rate (GFR) (mL/min/1.73 m^2^), mean ± SD	78.26 ± 2.46
Total cholesterol (mmol/L), mean ± SD	4.73 ± 0.26
Triglyceride (mmol/L), mean ± SD	1.23 ± 0.13
Low density lipoprotein (LDL) (mmol/L), mean ± SD	2.86 ± 0.24
High density lipoprotein (HDL) (mmol/L), mean ± SD	1.32 ± 0.89
LDL/HDL (mmol/L), mean ± SD	2.36 ± 0.31
Lactic acid (mmol/L), mean ± SD	2.87 ± 0.98
C-reactive protein (mg/L), mean ± SD	5.66 ± 2.54
White blood cell (×10^9^/L), mean ± SD	6.07 ± 0.76
GGC repeat size, mean (range)	115 (100–148)
**Cognitive function**	
MMSE Score, mean (range)	22 (15–27)
MoCA score, mean (range)	16 (14–21)
**Measurement of inclusion body fibers**	
Fibril length (nm), mean ± SD	167.91 ± 69.37
Fibril diameter (nm), mean ± SD	12.05 ± 5.47

SD, Standard Ddeviation; MELAS, Mitochondrial encephalomyopathy with lactic acidosis and stroke-like episodes; DWI, diffusion-weighted image; T2, T2-weighted image; MMSE, Minimum Mental State Examination; MoCA, Montreal Cognitive Assessment.

**Table 2:** Comparative analysis of clinical characteristics between the cognitively impaired group and the cognitively normal group of patients with NIID.

Cognitive Types in NIID Patients	With Cognitive Impairment N = 9	Without Cognitive Impairment N = 6	*p* Value
Age of onset (y), mean ± SD	61.33 ± 7.53	60.33 ± 8.43	0.814
Disease duration, mean y (range)	4 (3, 11)	4 (1.75, 11)	0.881
GGC repeat sizes, times (range)	115 (97.5, 122.5)	118.5 (97.25, 151.25)	0.890
Limb weakness, *n* (%)	1/9 (11.1%)	2/6 (33.3%)	0.692
Tremor, *n* (%)	6/9 (66.6%)	2/6 (33.3%)	0.459
Gait disturbance, *n* (%)	4/9 (44.4%)	4/6 (66.6%)	0.751
Abnormal behavior, *n* (%)	2/9 (22.2%)	1/6 (16.6%)	0.998
Headaches, *n* (%)	6/9 (66.6%)	2/6 (33.3%)	0.459
Encephalitis-like seizure, *n* (%)	5/9 (55.5%)	0/6 (0%)	**0.093**
MELAS-like episode, *n* (%)	1/9 (11.1%)	0/6 (0%)	0.996
Epileptic episode, *n* (%)	3/9 (33.3%)	1/6 (16.6%)	0.905
Autonomic dysfunction, *n* (%)	8/9 (88.8%)	6/6 (100%)	0.989
Sensory impairment, *n* (%)	2/9 (22.2%)	4/6 (66.6%)	0.236
Diabetes, *n* (%)	3/9 (33.3%)	0/6 (0%)	0.356
DWI high signal at dermatomedullary junction, *n* (%)	9/9 (100%)	5/6 (83.3%)	0.832
T2 high signal in corpus callosum, *n* (%)	8/9 (88.8%)	6/6 (100%)	0.997
T2 high signal of the external capsule, *n* (%)	8/9 (88.8%)	3/6 (50%)	0.283
Inner capsule T2 high signal, *n* (%)	0/9 (0%)	2/6 (33.3%)	0.277
Striatum T2 high signa, *n* (%)	0/9 (0%)	1/6 (16.6%)	0.832
T2 high signal in thalamus, *n* (%)	3/9 (33.3%)	2/6 (33.3%)	0.997
T2 high signal in brainstem, *n* (%)	5/9 (55.5%)	4/6 (66.6%)	0.989
T2 high signal in the middle cerebellar peduncle, *n* (%)	2/9 (22.2%)	2/6 (33.3%)	0.991
T2 high signal in the cerebellum, *n* (%)	5/9 (55.5%)	2/6 (33.3%)	0.751
T2 high signal in the centrum semiovale, *n* (%)	9/9 (100%)	5/6 (83.3%)	0.832
Lateral ventricle enlargement, *n* (%)	8/9(88.8%)	6/6 (100%)	0.989
Cerebellar atrophy, *n* (%)	8/9 (88.8%)	5/6 (83.3%)	0.997
Glomerular filtration rate (GFR) (mL/min/1.73 m^2^), mean ± SD	79.55 ± 13.59	76.33 ± 14.22	0.666
White matter lesion volume (cm^3^), mean ± SD	91.96 ± 42.60	87.25 ± 16.17	0.769
Total cholesterol (mmol/L), mean ± SD	4.58 ± 0.55	4.96 ± 1.55	0.505
Triglyceride (mmol/L), mean ± SD	1.33 ± 0.56	1.08 ± 0.46	0.370
Low density lipoprotein (LDL) (mmol/L), mean ± SD	2.72 ± 0.30	3.07 ± 1.49	0.496
High density lipoprotein (HDL) (mmol/L), mean ± SD	1.27 ± 0.36	1.39 ± 0.33	0.522
LDL/HDL (mmol/L), mean ± SD	2.29 ± 0.70	2.46 ± 1.83	0.803
Lactic acid (mmol/L), mean ± SD	2.14 ± 1.08	4.02 ± 6.02	0.484
C-reactive protein (mg/L), mean ± SD	8.73 ± 11.93	1.03 ± 0.93	**0.089**
White blood cell (× 10^9^/L), mean ± SD	6.79 ± 3.64	5.01 ± 1.01	0.196
Fibril length (nm), mean ± SD	143.47 ± 72.39	203.28 ± 50.16	**0.081**
Fibril diameter (nm), mean ± SD	14.27 ± 5.93	8.48 ± 1.51	**0.020**

Note: When the theoretical frequency is less than 5, the analysis is performed using fisher’s exact probability method.

**Table 3:** Comparison of demographic and clinical features of two groups of NIID patients with different types of fibril morphology.

Type of Fibril	Type A	Type B	*p* Value
Population proportion, *n* (%)	11 (73.3)	4 (26.7)	
Age of onset (y), mean ± SD	58.91 ± 7.68	67 ± 3.74	0.069
Disease duration, mean y (range)	4 (3–11)	4.5 (1.75–8)	0.949
GGC repeat sizes, times (range)	111 (100–137)	118 (94.75–187.5)	0.546
WML volume (cm^3^), mean ± SD	83.61 ± 22.77	106.31 ± 54.17	0.242
MoCA, score (range)	18 (16–23)	14 (11.75–14.75)	**0.039**
MMSE, score (range)	23 (21–28)	15 (14.25–15.75)	**0.016**
Fibril length (nm), mean ± SD	202.38 ± 42.35	73.08 ± 11.56	**0.006**
Fibril diameter (nm), mean ± SD	9.23 ± 2.21	19.79 ± 3.77	**0.004**
Fibril morphology	Long strip shape	Short rod-like	

Abbreviations: MMSE: Mini Mental State Examination, MoCA: Montreal Cognitive Assessment, WML white matter lesion.

## Data Availability

The datasets analyzed in this study are available from the corresponding author on reasonable request. The data are not publicly available due to legal or ethical reasons.

## Abbreviations

The following abbreviations are used in this manuscript:

NIID	Neuronal intranuclear inclusion disease
MMSE	Mini Mental State Examination
MoCA	Montreal Cognitive Assessment
WML	white matter lesion
H&E	hematoxylin and eosin
DWI	diffusion-weighted imaging
T2WI	T2-weighted imaging
FLAIR	fluid-attenuated inversion recovery
